# Optimal Design for 3-PSS Flexible Parallel Micromanipulator Based on Kinematic and Dynamic Characteristics

**DOI:** 10.3390/mi13091457

**Published:** 2022-09-02

**Authors:** Jun Ren, Qiliang Li, Hanhai Wu, Qiuyu Cao

**Affiliations:** School of Mechanical Engineering, Hubei University of Technology, Wuhan 430068, China

**Keywords:** flexible parallel mechanism, 3-PSS, optimal design, kinematics, dynamics

## Abstract

This paper proposes two optimal design schemes for improving the kinematic and dynamic performance of the 3-PSS flexible parallel micromanipulator according to different application requirements and conditions. Firstly, the workspace, dexterity, frequencies, and driving forces of the mechanism are successively analyzed. Then, a progressive optimization design is carried out, in which the scale parameters of this mechanism are firstly optimized to maximize the workspace, combining the constraints of the minimum global dexterity of the mechanism. Based on the optimized scale parameters, the minimum thickness and the cutting radius of the flexure spherical hinge are further optimized for minimizing the required driving forces, combined with constraints of the minimum first-order natural frequency of the mechanism and the maximum stress of the flexure spherical hinge during the movement of the mechanism. Afterward, a synchronous optimization design is proposed, in which the scale parameters are optimized to maximize the first-order natural frequency of the mechanism, combined with the constraints of a certain inscribed circle of the maximum cross-section of the workspace, the maximum stroke of the selected piezoelectric stages, and the maximum ultimate angular displacement of the flexure spherical hinge. The effectiveness of both optimization methods is verified by the comparison of the kinematic and dynamic characteristics of the original and optimized mechanism. The advantage of the progressive optimization method is that both the workspace and the driving forces are optimized and the minimum requirements for global dexterity and first-order natural frequency are ensured. The merit of the synchronous optimization method is that only the scale parameters of the mechanism need to be optimized without changing the structural parameters of the flexible spherical hinge.

## 1. Introduction

The flexible parallel micromanipulator transmits force and motion through the deformation of the flexure hinge [1,2,3] and combines a series of advantages of the parallel mechanism and flexible mechanism such as high load carrying capacity, high accuracy, no friction, no gap, easy assembly, and so on [4,5,6,7,8]. At present, it has a wide range of application prospects in the fields of microelectronics, microbial experiments, precision measurement, aerospace, and other fields [9,10,11,12].

In theoretical analysis and practical application, the optimal design is an effective means to improve the performance of a flexible parallel mechanism and expand its application fields [13,14]. The performance of a flexible parallel mechanism mainly refers to the kinematic characteristics (e.g., workspace and dexterity) and dynamic characteristics (e.g., natural frequency and driving force). Ding et al. [15] proposed a novel planar micromanipulation stage with large rotational displacement and obtained the kinematic model and reachable workspace of the platform analytically. Lu et al. [16] analyzed the kinematic characteristics of a 1-translational-3-rotational (1T3R) parallel manipulator, including a reachable position workspace and orientation workspace. The size and shape of the mechanism can be determined according to the given design index to meet the design requirements [17]. The optimal design of flexible parallel micromanipulators is mostly based on kinematic performance, such as workspace, dexterity, output/input displacement amplification ratio, etc. He et al. [18] optimized the dimensions of the TCP-actuated finger mechanism with the local and global performance concerning the dexterity and the extreme value of the velocity as the evaluation indices. Ding et al. [19] proposed an FEA-based optimization method based on structural parameters to solve the problem of insufficient constant-force stroke of the compliant constant force mechanism (CFM) based on a flexible Z-shaped beam and a bistable beam. Yang et al. [20] proposed a 3-PRR-compliant parallel robot and optimized the geometric parameters of the mechanism and the flexure hinge by a genetic algorithm to obtain the desired motion performance. Xu et al. [21] established a kinematics model of a compliant mechanism with one flexible joint designed from a rigid four-bar linkage. They optimized the structural parameters of the mechanism by taking the path deviation and strain energy as two objectives. Li et al. [22] proposed a new 2-DOF-compliant micromanipulator and established a kinematics model. Then, kinematic optimization of the design parameters was carried out. Li et al. [23] also performed performance improvements and dimension optimizations on the 3-PRC-compliant parallel micromanipulator to improve several disadvantages of the mechanism in the aspects of stiffening, buckling, and parasitic motions. Xu et al. [24] carried out the optimal design of a 3-PUU flexible parallel micromanipulator by taking the maximum value of the mechanism’s workspace and the weighted combination of the mechanism’s dexterity as the optimization objective. Jia et al. [25] optimized the 3-PRR flexible parallel mechanisms with the workspace as the optimization objective and dexterity as the constraint condition. In addition to kinematic performance, a few scholars have also carried out research on the optimal design of flexible parallel mechanisms for dynamic performance. Wang et al. [26] proposed a compliant mechanisms optimization method based on dynamic characteristics and verified the feasibility of the optimization method based on a specific configuration. Li et al. [27] proposed a dynamics modeling and optimization method for a 2-DOF translational parallel robot with flexible links for a high-speed pick-and-place operation. The dimension of the mechanism can be optimized according to sensitivity and dynamic stress to improve the dynamic accuracy of the end-effector at high speed. Du et al. [28] carried out the optimal design of the 3-RRR flexible parallel mechanism based on dynamic performance and obtained the optimal mechanism with lightweight and small deformation. Li et al. [29] proposed a class of an XY totally decoupled parallel stage and established the kinematics and dynamics model of the mechanisms. The stage structure optimization was then carried out to achieve a maximal natural frequency under the performance constraints such as workspace. After that, Li et al. [30] presented a novel compliant parallel XY micro-motion stage, and the dimensions of the mechanism were optimized for maximizing the natural frequencies. To sum up, current optimal designs of flexible parallel micromanipulators are mostly based on kinematic performance or dynamic performance, but few of them take into account both. In practical applications, however, it is sometimes necessary to consider both kinematics and dynamics characteristics to meet specific requirements.

In our previous studies [31], a novel 3-PSS flexible parallel micromanipulator was proposed, its dynamics model was established, and its frequency characteristics were analyzed. In order to optimize the performance of the mechanism to meet both the kinematic characteristics and dynamic characteristics, this paper proposes two optimization schemes for designing the 3-PSS flexible parallel micromanipulator. One of the schemes is called the progressive optimization strategy, in which the scale parameters of the mechanism are firstly optimized according to the kinematic performance requirement and then the structural parameters of the flexure spherical hinge are further optimized according to the dynamic characteristic requirements based on the optimized scale parameters. Another scheme is a synchronized optimization strategy, in which the scale parameters of the flexible parallel micromanipulator are optimized by taking the kinematic performance and the dynamic performance as the constraint condition and the optimization object, respectively. Since the range of motion of the flexible parallel mechanism is usually small, the workspace and motion accuracy (directly related to the dexterity of the mechanism) are usually the major consideration in the kinematic performance. In dynamic performance, the natural frequency of the structure and driving forces required are often concerned more. Therefore, the optimization of kinematic performance and dynamic performance mentioned in this paper mainly refers to the workspace, dexterity, natural frequencies, and driving forces.

## 2. Kinematics and Dynamics Performance Analysis

**Figure 1:**
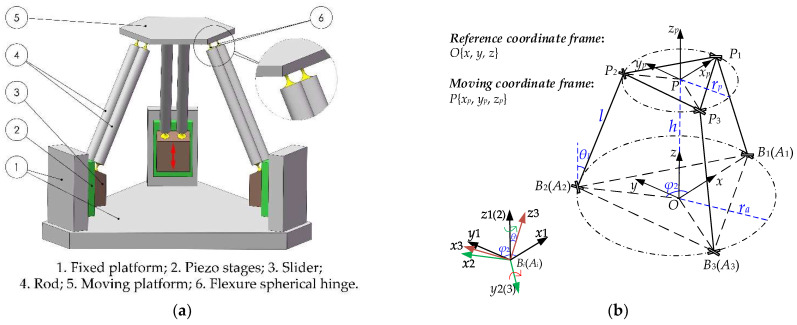
(**a**) 3D structure of 3-PSS flexible parallel micromanipulator; (**b**) simplified pseudo-rigid body model and coordinate frame settings.

The 3-PSS (P—prismatic pair; S—spherical joint) flexible parallel micromanipulator has three translational degrees of freedom in space, and its 3D structure is shown in Figure 1a. This mechanism is composed of a fixed platform, a moving platform, and three identical branches. Three branches connecting to the moving platform and the fixed platform of the mechanism are equally distributed around the moving platform by 120°. Each branch consists of a piezo stage, four flexure spherical hinges, and two rigid rods. Three translational DOFs of the moving platform can be achieved by coordinating up-and-down movements of the three piezo stages.

### 2.1. Kinematics Analysis of 3-PSS Flexible Parallel Micromanipulator

In order to simplify the analysis, the “simplified pseudo-rigid body model” of the 3-PSS flexible parallel micromanipulator is established, as shown in Figure 1b. The reference coordinate frame O{*x*, *y*, *z*} is set at the circumcircle center of the equilateral triangle formed by centroid *A_i_* (*i* = 1, 2, 3) of three sliders in the initial state, and the radius *r_a_* of the circle is defined as the radius of the fixed platform. Similarly, the moving coordinate system *P* {*x_p_*, *y_p_*, *z_p_*} is established at the center P of the moving platform, and the radius of the moving platform is defined as *r_p_*. The *x*-axis and *y*-axis of the two coordinate frames are parallel and their *z*-axis overlaps. In the initial state, the vertical distance between the moving platform and the fixed platform is *h*. The length of each link *B_i_P_i_* is *l*, where points *B_i_* and *P_i_*, respectively, represent the center points of the flexure spherical hinges at both ends of the link. For the convenience of analysis, it is assumed that the center of mass of the slider coincides with the center of mass of the flexible spherical hinge at the lower end of the connecting rod. Thus, *A_i_B_i_* represents the input displacement of the *i-*th slider. *OA_i_* is the position vector from the center point O of the stationary platform to the center point *A_i_* of the slider in the initial state, and *φ_i_* (*φ*_1_ = 0; *φ_i_*_+1_ = *φ_i_* + 2/3π) is the angle between *OA_i_* and the *x*-axis of the reference coordinate frame. The angle between the *B_i_P_i_* and the *z*-axis of the reference coordinate frame is defined as *θ_l_*, as shown in Figure 1b.

Workspace and dexterity are important indicators for evaluating the kinematic performance of a mechanism. Therefore, the kinematic model of the mechanism is established first and the workspace and dexterity characteristics of the micromanipulator are further analyzed. The position vector of the center point *P* of the moving platform in the reference coordinate frame *O*{*x*, *y, z*} is expressed as *P* = (*x*, *y*, *z*)^T^, as shown in Figure 1b. The closed vector equation of the *i*-th branch can then be established according to the closed-loop vector method.

(1) $$OA_{i}+A_{i}B_{i}=OP+PP_{i}+P_{i}B_{i}$$

The inverse kinematics equation of the micromanipulator can be obtained by arranging Equation (1) as follows:

(2) $$d_{i}=z-\sqrt{l^{2}-{\left(x-E_{r}\cos\varphi_{i}\right)}^{2}-{\left(y-E_{r}\sin\varphi_{i}\right)}^{2}}$$

where *d_i_* is the displacement of the *i-*th slider.

The relationship between the input and output of the micromanipulator can be obtained by taking the derivative of Equation (2) with respect to time.

(3) $$\dot{B}=J\dot{P}$$

where ***J*** is the velocity Jacobian matrix of the micromanipulator. $\dot{B}\;$ and $\dot{P}$ are the velocities of the sliders and the moving platform, respectively.

#### 2.1.1. Workspace Analysis of 3-PSS Flexible Parallel Micromanipulator

The workspace of the flexible parallel micromanipulator refers to the working area that can be achieved by the center *P* of the moving platform, which is an important indicator for evaluating the motion performance of the micromanipulator. Here we choose the same set of parameters as in the literature [31], as listed in Table 1.

During the working process of the micromanipulator, the angular displacement of the flexure spherical hinge will change with the change of the position of the moving platform. However, the angular displacements of the flexible spherical hinges on the same branch are always the same. Hence, the angular displacement of the flexure spherical hinge on the *i*-th branch can be expressed as:

(4) $$\psi_{i}=\frac{n_{i}^{0}\cdot n_{i}}{\left|n_{i}^{0}\right|\left|n_{i}\right|}$$

where, *i* = 1, 2, 3 represents the *i*-th branch, $n_{i}^{0}$ represents the axial direction vector of the rod in the initial state, and ***n****_i_* represents the axial direction vector of the rod at any time.

In order to avoid the fracture of the flexure spherical hinge due to excessive angular displacement, the following constraint is established:

(5) $$0\leq \psi_{i}\leq \psi_{\max}$$

where $\psi_{\max}$ is the maximum ultimate angular displacement of the flexure spherical hinge, which is assumed to be 1°.

The maximum displacement *d*_max_ of the selected piezoelectric stages is 200 μm, and the following constraint is established for the slider displacement *d_i_*.

(6) $$0\leq d_{i}\leq d_{\max}$$

**Figure 2:**
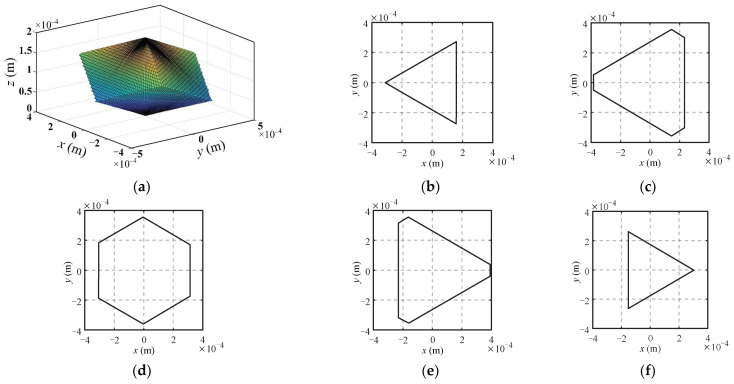
The workspace of the 3-PSS flexible parallel micromanipulator and sectional shapes of workspace at different heights. (**a**) The workspace; (**b**) *z* = 50 μm; (**c**) *z* = 75 μm; (**d**) *z* = 100 μm; (**e**) *z* = 125 μm; (**f**) *z* = 150 μm.

Based on the above constraints, the workspace of the micromanipulator can be calculated by employing the cylindrical limit search method [25] with the parameters given in Table 1, as shown in Figure 2a. It is shown that the workspace of the mechanism is a closed symmetrical shape, the total height *z_max_* of the workspace is 200 μm, which is equal to the stroke of the slider *d_max_*. Sectional shapes of the workspace at different heights are also provided in Figure 2c–f. It can be seen that the maximum cross-section of the workspace is on the plane at the height *z_max_*/2, that is 100 μm.

In order to quantify the size of the workspace, a cube covering the entire workspace of the micromanipulator is selected with a volume *V* which is evenly divided into *N* units. Then we calculate the angular displacements of the flexible spherical hinge and the displacements of the slider when the center of the mobile platform is located at the center of each unit. If the angular displacements of the flexure spherical hinge and the stroke of the driver satisfy the constraints, the unit is reserved, and the total number of reserved units is denoted as *n*. Thus, the volume *V_w_* of the workspace can be expressed as:

(7) $$V_{w}=\frac{n}{N}V$$

**Figure 3:**
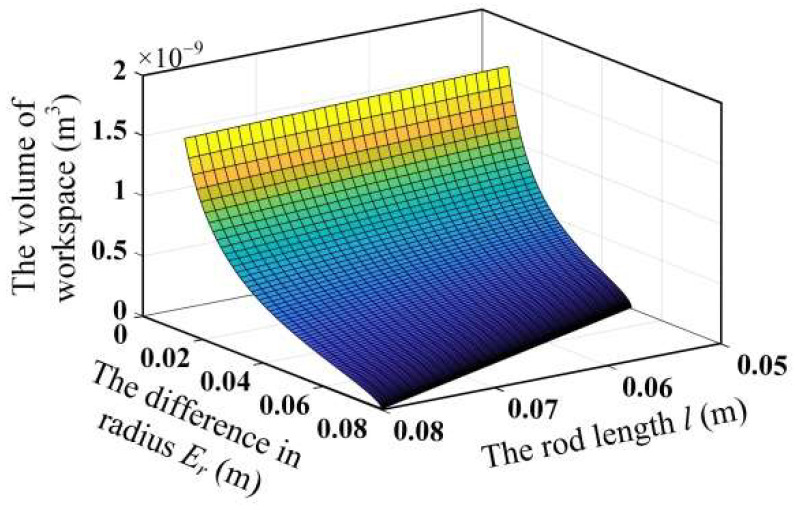
Relationship between workspace volume and scale parameters.

To clarify the influence of the scale parameters on the workspace volume of the mechanism, the variation of the workspace volume with the scale parameters is obtained by changing the rod length *l* of the mechanism and the difference between the moving and fixed platform radius *E_r_*, as shown in Figure 3. It can be seen that the workspace volume of the mechanism increases with the increase in the rod length *l* and decreases with the increase in the difference in radius *E_r_*.

#### 2.1.2. Dexterity Analysis of 3-PSS Flexible Parallel Micromanipulator

**Figure 4:**
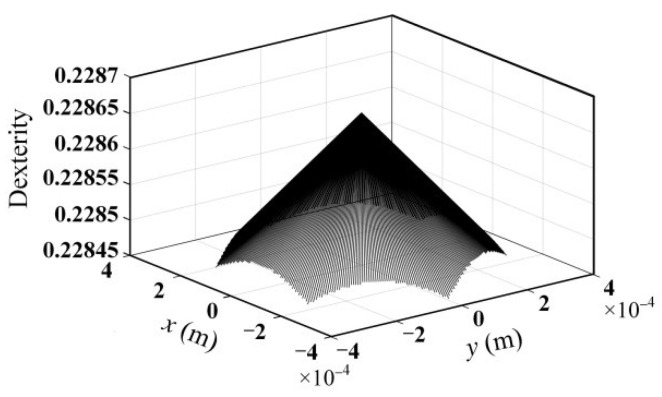
Dexterity within the maximum cross-section of the workspace of the mechanism.

Dexterity is an important kinematic property of the flexible parallel micromanipulator. The general Jacobian matrix condition number *k* is used as a measure of the dexterity of the mechanism, where $k=\left\|J\right\|•\left\|J^{-1}\right\|$, ‖•‖ represents the two-norm of the matrix. Furthermore, the dexterity of the mechanism is usually represented by the inverse of the Jacobian matrix condition number, that is *u* = 1/*k*. When *u* = 0, the mechanism is in odd isotropy. When *u* = 1, the mechanism is in isotropy. According to the mechanism model parameters given in Table 1, the dexterity distribution on the maximum cross-section (*z* = *z_max_*/2) in a micromanipulator’s workspace can be obtained, as shown in Figure 4.

According to the literature [24], the global dexterity in the workspace of the mechanism can be expressed as:

(8) $$GDI\approx \frac{1}{N_{w}}\sum_{w\in N_{w}}\frac{1}{k}$$

where *w* is one of the *N_w_* points uniformly distributed in the workspace. With the dimension parameters given in Table 1, the calculation result of the global dexterity in the workspace of the mechanism is 0.2287.

**Figure 5:**
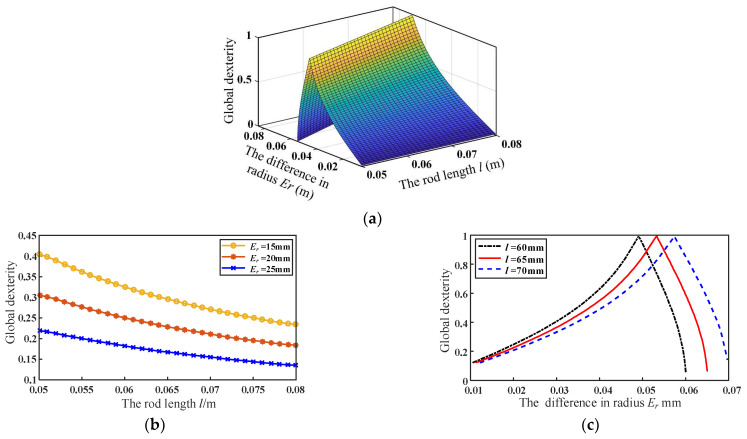
Relationship between scale parameters and global dexterity: (**a**) the effect of rod length *l* and radius difference *E_r_* on global dexterity; (**b**) the effect of rod length *l* on global dexterity; (**c**) the effect of radius difference *E_r_* on global dexterity.

To clarify, the effect of the scale parameters on the global dexterity of the mechanism, the radius difference *E_r_*, and the rod length *l* were varied, respectively. Then the variation of the global dexterity of the mechanism with the scale parameters can be obtained, as shown in Figure 5a. It can be seen from Figure 5b that the global dexterity of the mechanism decreases with the increase in rod length. Furthermore, it first increases to 1 and then decreases during the increase in the radius difference *E_r_*, as shown in Figure 5c.

### 2.2. Dynamics Analysis of 3-PSS Flexible Parallel Micromanipulator

Dynamic analysis is a necessary way to acquire the dynamic performance of a micromanipulator, including the relationship between the motion trajectory of the moving platform and the driving forces and natural frequency characteristics. The dynamic model of the mechanism is established by utilizing the Lagrange equation method. According to the dynamic model of the 3-PSS flexible parallel micromanipulation mechanism [31], the dynamic equation of the mechanism is expressed as:

(9) $$M\ddot{s}+Ks+G=F$$

where ***M*** is the mass matrix of the mechanism, ***K*** is the stiffness matrix of the mechanism, ***G*** is the gravity matrix of the mechanism, ***s*** is the displacement of the moving platform, and ***F*** is the generalized force exerted on the moving platform.

According to Equation (9), the undamped natural frequency of the mechanism can be determined by:

(10) $$\left|K-\omega^{2}M\right|=0$$

where *ω* represents the circular frequency of the mechanism, and the natural frequency of the mechanism is *f* = *ω*/(2*π*).

According to the virtual work principle, the driving force of the sliders is computed as:

(11) $$F_{b}=J^{-T}F$$

where ***J*** is the velocity Jacobian matrix of the mechanism.

**Figure 6:**
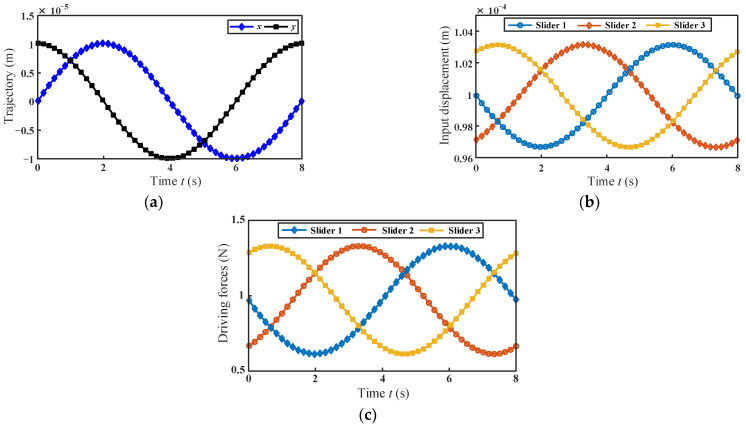
(**a**) Movement trajectory of the moving platform; (**b**) displacements of the sliders; (**c**) the driving forces of the micromanipulator.

The motion trajectory of the moving platform is selected within the maximum cross-section of the workspace. The trajectory equation is given in Equation (12), and the driving displacement of the sliders can be obtained according to Equation (2). The motion trajectory of the moving platform and the corresponding driving displacements of the sliders are shown in Figure 6a,b, respectively.

(12) $$\left\{ \begin{array}{l} x=1\times 10^{-5}\sin(\omega t)\\ y=1\times 10^{-5}\cos(\omega t)\\ z=1\times 10^{-4} \end{array}\right.$$

where ω = π/4.

With the given parameters listed in Table 1, the driving force required by each branch can be obtained. As shown in Figure 6c, the maximum absolute value *F_bM_* of the driving force on each branch is identical, and the driving force on each branch of the mechanism changes regularly. By comparing Figure 6b,c, it can be seen that the variations of the driving forces are consistent with the variations of the input displacements of the branches.

**Figure 7:**
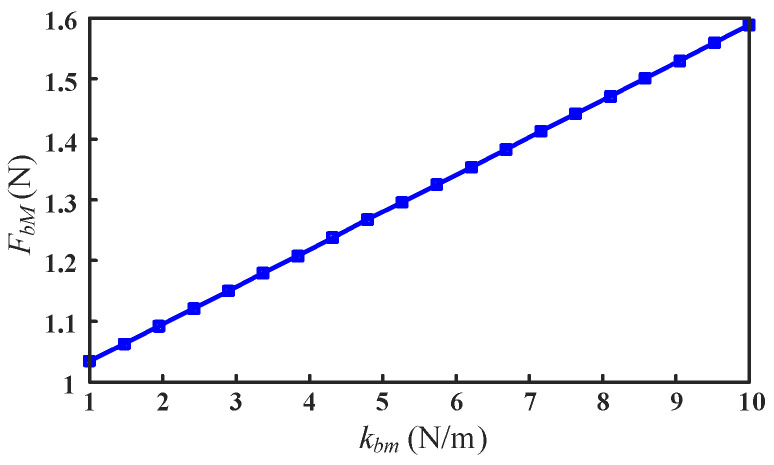
Influence of the bending stiffness of the flexure spherical hinge on the driving force of the mechanism.

According to Equations (9) and (11), it can be seen that the driving forces $F_{b}$ of the sliders are related to the mass, stiffness of the mechanism, and the motion trajectory of the moving platform. For simplicity, the maximum absolute value of the driving force is used as the evaluation index to analyze the influence of the mass and stiffness of the mechanism on the driving forces under the condition of a given platform motion trajectory. According to the configuration and motion characteristics of the mechanism, the stiffness that affects the driving force $F_{b}$ is mainly derived from the bending stiffness of the flexible spherical hinge. Therefore, we analyzed the variation of the driving force by changing the bending stiffness *k_bm_* of the flexure spherical hinge. As shown in Figure 7, with the increase in the bending stiffness *k_bm_* of the flexure spherical hinge, the maximum absolute value *F_bM_* of the driving force increases linearly.

**Figure 8:**
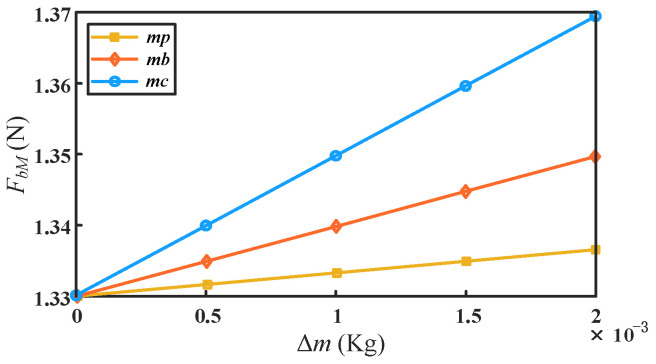
Influence of absolute mass change of different components on driving force.

On the other hand, the influence of the mass change of different components of the mechanism on the driving force can be obtained by individually increasing the mass of the main components according to Equations (9) and (11). As shown in Figure 8, the maximum absolute value of the driving force of the mechanism also increases as the mass of the components increases. When increasing the same absolute mass Δ*m*, the variation of the rod mass *m_c_* has the greatest influence on the driving force, followed by the slider mass *m_b_*. The mass *m_p_* of the moving platform has the least influence on it.

## 3. Progressive Optimal Design Based on Kinematics and Dynamics

**Figure 9:**
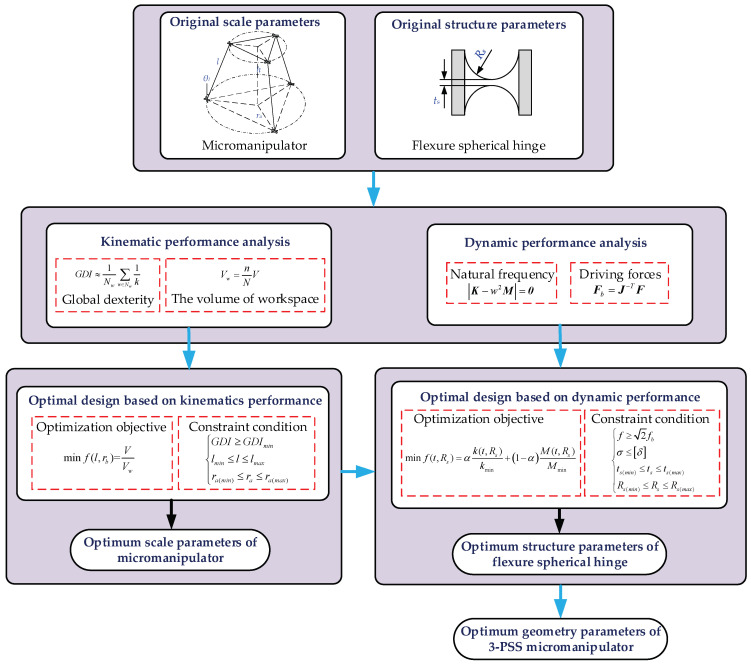
The progressive optimization procedure for analyzing and designing 3-PSS flexible parallel micromanipulator.

According to the analysis in the previous section, the kinematics and dynamic performance of the 3-PSS flexible parallel micromanipulator can be obtained. In order to make the mechanism meet the requirements involving certain kinematics and dynamic performance at the same time, a progressive optimization design is proposed. First, the scale parameters of the mechanism are optimized with the kinematic performance as the optimization objective. Based on the optimized scale parameters, the structural parameters of the flexure spherical hinge are further optimized by taking the dynamic performance as the optimization objective. The optimization process is shown in Figure 9.

### 3.1. The Scale Parameters Optimization Based on Kinematic Performance

It can be known from the kinematic Equation (2) that the kinematic performance is mainly related to the rod length *l* and the difference between the moving and fixed platform radius *E_r_*. In order to simplify the analysis, the radius of the moving platform of the mechanism is assumed to be a constant, and the fixed platform radius *r_a_* and the rod length *l* are used as the optimization parameters.

For the convenience of optimization, the variation range of the rod length *l* is given as approximately ±20% of the original size. The corresponding variation range of the radius *r_a_* of the fixed platform can be obtained through the variation range of the rod length *l* and the geometry relationship of the mechanism. It is assumed that the stroke *d_max_* of the selected piezoelectric stages is 200 μm, and the ultimate angular displacement $\psi_{\max}$ of the flexure spherical hinge is 1°. When designing a flexible parallel mechanism, higher motion accuracy and a larger workspace are usually expected, but the two are contradictory. Since the motion accuracy is related to global dexterity, we take the minimum global dexterity (assumed to be 0.2 in this study) as one of the constraints, the minimum ratio of the mechanism’s volume to the workspace volume as the objective function, and then combine the constraints of the scale parameters (*l* and *r_a_*) to formulate the following optimization model.

(13) $$\min f(l,r_{a})=\frac{V}{V_{w}}$$

(14) $$\left\{ \begin{array}{l} GDI\geq 0.2\\ 50\;\mathrm{mm}\leq l\leq 80\;\mathrm{mm}\\ 26\;\mathrm{mm}\leq r_{a}\leq 105\;\mathrm{mm} \end{array}\right.$$

where *V_w_* represents the volume of the workspace of the micromanipulator and *V* represents the volume of the workspace of a cube covering the entire workspace of micromanipulator.

According to the above constraints and optimization objective, optimization is carried out by employing the genetic algorithm toolbox in MATLAB R2018b software (MathWorks, Natick, MA, USA). The optimized scale parameters are *r_a_* = 38.66 mm and *l* = 50.13 mm, respectively. The global dexterity GDI of the optimized mechanism is 0.204, which meets the design requirements.

**Figure 10:**
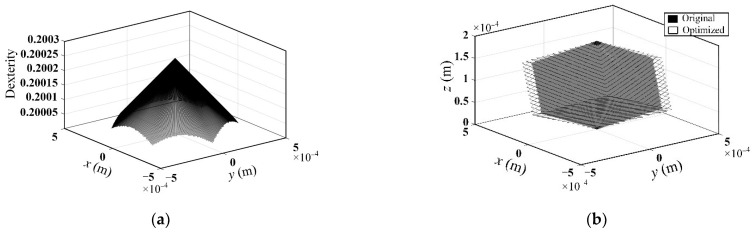
(**a**) The dexterity within the maximum cross-section of the workspace of the optimized mechanism; (**b**) workspace comparison between original and optimized designs.

The dexterity distribution of the optimized mechanism on the maximum cross-section in the workspace is shown in Figure 10a. It can be seen that maximum dexterity occurs at the center of the maximum cross-section. When the position of the moving platform changes, the dexterity changes accordingly, and the dexterity gradually decreases along the direction away from the center of the maximum cross-section. The comparison of the workspace volume between original and optimized designs is shown in Figure 10b. It is demonstrated that the workspace volume of the optimized mechanism has increased by 14.17% compared with the original design. It is indicated that the optimal design of mechanism scale parameters based on kinematic performance is effective.

### 3.2. Optimization of Flexure Spherical Hinge Structure Parameters Based on Dynamic Performance

**Figure 11:**
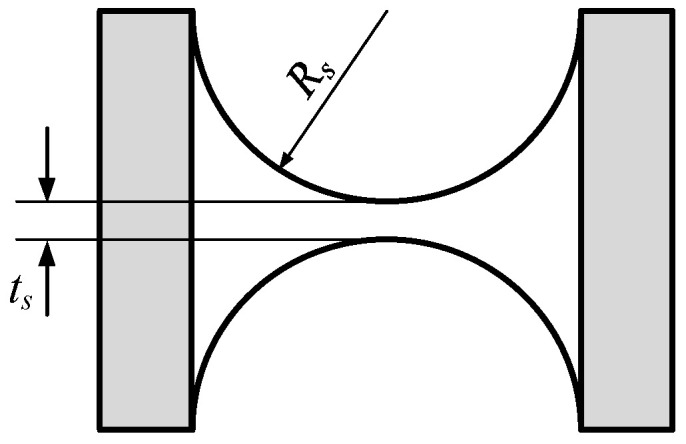
The schematic diagram of flexure spherical hinge structure.

The scale parameters that meet the requirements of the kinematic performance of the micromanipulator were obtained through the above optimization design. However, the dynamic performance of the mechanism is not considered, which has an important impact on the high-frequency control scheme of the mechanism and the selection of the driver. Results from the related research [31] show that the dynamic performance of the flexible parallel micromanipulation mechanism is related to the micromanipulator’s scale parameters as well as the structural parameters of the flexure spherical hinge. Among them, the structural parameters of the flexure spherical hinge are the main factors affecting the dynamic performance of the micromanipulator. Therefore, the structural parameters of the flexure spherical hinge are chosen to further optimize its dynamic performance. The flexible hinge of the 3-PSS flexible parallel micromanipulator is a right-circular flexure spherical hinge, and its structural diagram is shown in Figure 11. The main parameters of the flexible hinge are the minimum thickness *t_s_* and the cutting radius *R_s_*. Therefore, the minimum thickness *t_s_* and the cutting radius *R_s_* are selected as the optimization parameters.

The natural frequency is required to be greater than $\sqrt{2}$ times the fundamental frequency *f*_b_ of the driver (piezo stage) for preventing resonance, and *f*_b_ of the selected piezo stage is 37 Hz in this study. Meanwhile, the maximum stress $\sigma_{\max}$ of the flexure spherical hinge during the movement of the mechanism should be less than the permissible stress [σ] so as to prevent fatigue fracture. Considering the requirements of machining and deformation of the flexible spherical hinge, certain size ranges (unit: mm) are given for the structural parameters *t_s_* and *R_s_*. In summary, the constraint expression for the optimal design can be written as:

(15) $$\{\begin{array}{l} \;f\;\;\geq \sqrt{2}f_{b}\\ \begin{array}{l} \;\sigma_{\max}\;\;\leq \left[\sigma \right]\\ 0.8\leq \;\;t_{s}\leq 1.2\\ \;\;1\;\;\leq R_{s}\leq 10 \end{array} \end{array}$$

The driving force is an important dynamic performance of the micromanipulator and is therefore chosen as the optimization objective. It can be seen from the previous section that the driving force is mainly related to the total mass and stiffness of the mechanism. Generally, the driving force of the micromanipulator is smaller as the mass of the mechanism decreases. For brevity, the mass of the moving platform is set as a constant value, and the mass of the rod and the slider are set to be variable. Since the scale parameters have been determined according to the previous kinematic optimization results, the mass of the rod is only related to the diameter *D* of the rod. According to the 3D structure shown in Figure 1a, the diameter *D* of the rod is required to be not less than the sum of the minimum thickness *t_s_* and twice the cutting radius *R_s_* of the flexure spherical hinge. Here we assume that the diameter of rod *D* = *t_s_* + 2*R_s_* + 2 mm. In addition, the size of the slider is also directly related to the diameter of the rod *D*. In summary, the total mass of the micromanipulator is mainly related to the structural parameters of the flexure spherical hinge. It can be seen from Equations (9) and (11) that under the same motion requirement, the smaller the total mass of the mechanism, the smaller the driving force required. Hence, the minimum total mass *M* of the mechanism is taken as one of the optimization objectives.

It is known from previous analysis that the bending stiffness *k_bm_* of the flexure spherical hinge directly affects the overall stiffness of the mechanism, and further affects the driving force and natural frequencies of the micromanipulator. From the analysis of driving force characteristics in Section 2.2, it can be seen that the less the stiffness of the flexure spherical hinge, the less the required driving force. Therefore, the minimum stiffness is chosen as another optimization objective.

Taking the above two optimization objectives into consideration, in order to make the required driving force small enough, a minimum weighted combination of the total mass of the mechanism and the bending stiffness of the flexible spherical hinge is selected as the overall optimization objective. The overall optimization objective function can be constructed as:

(16) $$\min f(t,R_{s})=\alpha \frac{k(t,R_{s})}{k_{\min}}+\left(1-\alpha \right)\frac{M(t,R_{s})}{M_{\min}}$$

where the weight factor *α* (*α* ∈ [0, 1]) represents the proportion of bending stiffness in the optimization. In order to make the two optimization objectives (*k* and *M*) in the same order of magnitude, they are divided by *k_min_* and *M_min_*, respectively, which represent the minimum values of bending stiffness and overall mass under the constraint conditions, respectively.

Governing the constraints in Equation (15) and the optimization objective in Equation (16), optimization is carried out by employing the genetic algorithm toolbox in MATLAB R2018b software. It should be noted that the optimization results of the structural parameters of the flexure spherical hinge are different under different weight factors. Taking the motion trajectory of Equation (17) as an example, we perform the optimization. In order to obtain the structural parameters of the flexure spherical hinge resulting in the smallest maximum absolute value of the driving force, the optimization results of structural parameters of the flexible spherical hinge under different weight factors are first obtained successively. Then we calculate the maximum absolute values *F_bM_* of the driving force corresponding to each set of structural parameters. Relevant results are collected in Table 2.

(17) $$\left\{ \begin{array}{l} x=2\times 10^{-5}\sin(\omega t)\\ y=2\times 10^{-5}\cos(\omega t)\\ z=1\times 10^{-4} \end{array}\right.$$

where *ω* = π/4.

It can be seen from Table 2 that the maximum absolute value *F_bM_* of the driving force first decreases, then increases, and then remains unchanged as the stiffness weight factor *α* increases with an interval of 0.1. After the weight factor is increased to 0.5, as it continues to increase, the optimization results remain unchanged. This is because when the weight *α* is increased to 0.5, some parameters or properties of the optimized mechanism reach critical values of constraints, so continuing to increase *α* will not change the optimization results. It is easy to find that the maximum absolute value *F_bM_* of the driving force reached the smallest value (1.7303 N) when the weight factor *α* is 0.3. However, the smallest value is very close to the maximum absolute value (1.7304 N) when the weight factor *α* is 0.2. Therefore, in order to obtain a more ideal optimization model, another group of weight factors is selected between 0.2–0.3 with the interval of 0.01, and further optimization is carried out according to the above method. Results in Table 3 show that the smallest maximum absolute value (1.7223 N) of the driving force appears as the weight factor α is 0.25. Correspondingly, the structure parameters of the flexure spherical hinge obtained by optimization are *t_s_* = 0.8 mm and *R_s_* = 1.829 mm. In addition, it is interesting to note that no matter the weight factor, *α* takes any value from 0.1 to 0.9, the optimization results of *t_s_* always coincide with the minimum value in the constraints, that is 0.8. It demonstrates that the minimum thickness *t_s_* of the flexible hinge has a larger impact on the driving forces compared to the cutting radius *R_s_*.

**Figure 12:**
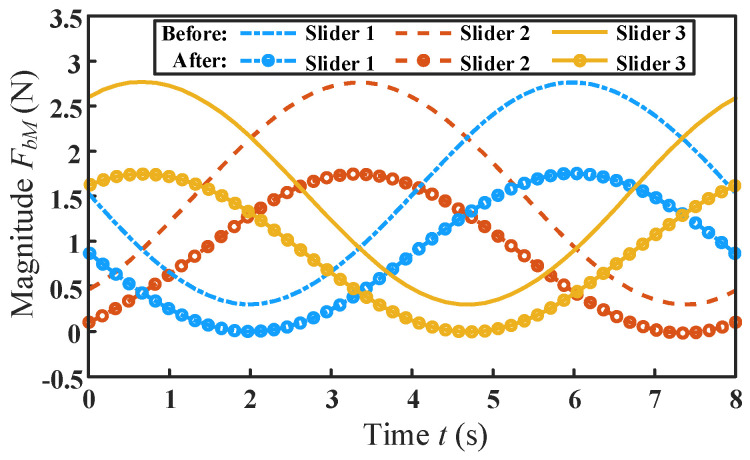
The comparison of driving forces before and after optimization.

The comparison of driving forces before and after optimization is shown in Figure 12. It can be seen that the variation trend of the driving forces before and after optimization is consistent. After optimization, the driving forces are decreased to different degrees on the entire time axis. Moreover, the maximum absolute value *F_bM_* of driving forces is reduced by 34.54% compared with that before optimization.

The structural parameters of the flexure spherical hinge and the dynamic performance of the mechanism before and after optimization are shown in Table 4. It can be seen from the comparison that the overall mass of the mechanism is reduced by 8.34% after optimization, which greatly reduces the inertial force of the mechanism. At the same time, it is also found that the stiffness and first-order natural frequency of the mechanism are reduced to a certain extent, but they are all within the allowable range. In addition, the ultimate angular displacement of the optimized flexure spherical hinge is slightly reduced, but it is still much larger than the initial set value of 1°. In conclusion, optimization based on dynamic performance is feasible and effective. It can be seen that when the acceleration in Equation (9) is 0, the dynamic equation is transformed into a static equation. Thus, the optimization method is also suitable for optimization design based on a quasi-static performance.

## 4. Synchronous Optimal Design Based on Kinematics and Dynamics

**Figure 13:**
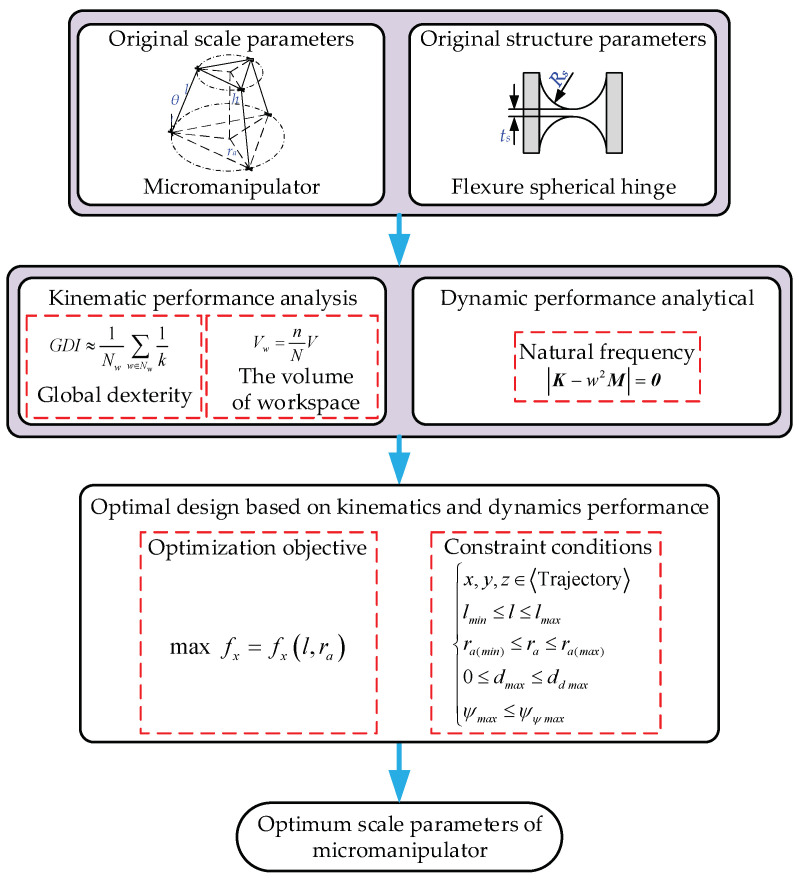
The synchronous optimization procedure based on kinematics and dynamics performance.

In practical applications, the manufacturing difficulty of flexible spherical hinges is usually much greater than that of other structural parts, and especially the minimum thickness of the spherical hinge is often less than 1 mm, which is often easy to damage by processing. Therefore, when improving the existing 3-PSS flexible parallel micromanipulator to optimize its kinematics and dynamics, sometimes it is not desirable to modify the flexible spherical hinge, and only the scale parameters of the mechanism can be optimized. The following proposes a method to optimize the scale parameters of the mechanism based on both kinematic performance and dynamic performance. In this optimization method, the first-order frequency of the mechanism is used as the optimization objective and the inscribed circle radius at the maximum cross-section of the workspace is selected as constraints. The optimization procedure is shown in Figure 13.

Similar to Section 3.1, the moving platform radius *r_p_* of the mechanism is set to be a constant, and the fixed platform radius *r_a_* and the rod length *l* are used as the optimization parameters. The variation range of the rod length *l* is selected as approximately ±20% of the original size. The corresponding variation range of the radius *r_a_* of the fixed platform can be obtained through the variation range of the rod length *l* and the geometry relationship of the mechanism. Considering the maximum cross-section of the workspace is a polygon (see Figure 2d), the workspace of the mechanism is measured by the radius of the inscribed circle in the maximum cross-section. In this study, the radius of the inscribed circle of the maximum cross-section of the workspace is selected to be 400 μm. It is assumed that the maximum stroke *d_max_* of the selected piezoelectric stages does not exceed 200 μm, and the ultimate angular displacement $\psi_{\max}$ of the flexure spherical hinge does not exceed 1°. Then the constraint condition can be combined as:

(18) $$\left\{ \begin{array}{l} x=4\times 10^{-4}\sin(\omega t),\;y=4\times 10^{-4}\cos(\omega t),\;z=1\times 10^{-4}\\ 50\;\mathrm{mm}\leq l\leq 80\;\mathrm{mm}\\ 26\;\mathrm{mm}\leq r_{a}\leq 105\;\mathrm{mm}\\ 0\leq d_{\max}\leq 200\;\mu m\\ \psi_{\max}\leq 1^{{}^{\circ}} \end{array}\right.$$

where *ω* = π/4.

According to the symmetry of the micromanipulator’s structure, the first and second-order natural frequencies (corresponding to the motion in the *x* and *y* directions, respectively) of the mechanism are equal. Hence, the natural frequency *f_x_* corresponding to the motion in the *x* direction (or the *y* direction) is selected as the optimization objective. The optimization objective function is established as:

(19) $$\max\;f_{x}=f_{x}\left(l,r_{a}\right)$$

According to the above optimization parameters (*l* and *r_a_*), constraints (Equation (18)), and optimization objective (Equation (19)), optimization can thus be carried out by employing the genetic algorithm toolbox in MATLAB R2018b software. The optimized scale parameters are *l* = 50 mm and *r_a_* = 36.92 mm, respectively. Correspondingly, the natural frequency (*x* or *y* direction) of the optimized mechanism is 73.61 Hz.

**Figure 14:**
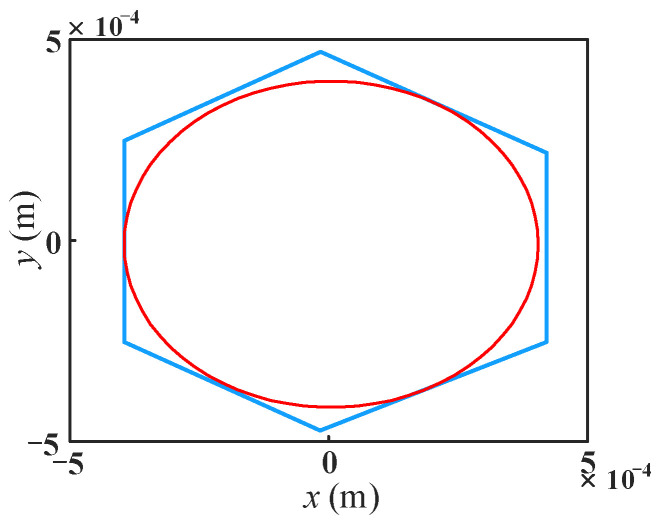
Maximum cross-section of the workspace of optimized mechanism.

**Figure 15:**
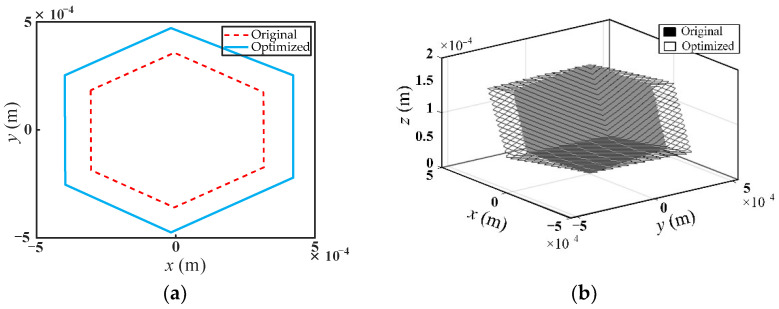
Comparison of the workspace between original and optimized mechanism, (**a**) the maximum cross-section of the workspace of the mechanism; (**b**) micromanipulator workspace.

In order to verify whether the optimization results meet the constraints, the workspace analysis of the optimized mechanism is carried out. As shown in Figure 14, the maximum inscribed circle radius on the maximum cross-section of the workspace of the optimized mechanism is 400 μm, which satisfies the optimization constraints. The comparison of the workspace of the original and optimized mechanisms is given in Figure 15. As can be seen from Figure 15a, the maximum cross-sectional shape of the optimized mechanism’s workspace remains a regular hexagon compared with the original mechanism, but the area is increased. The workspace volume of the optimized mechanism increases by 31.93% compared with that of the original mechanism, as shown in Figure 15b.

**Figure 16:**
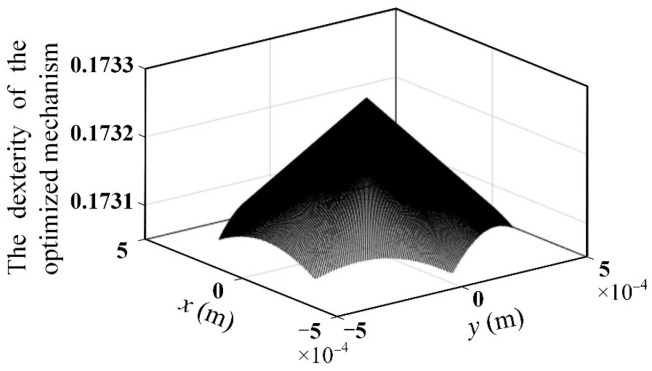
The distribution of dexterity within the maximum cross-section of the workspace of the optimized mechanism.

Since the dexterity of the mechanism directly reflects the motion accuracy of the mechanism, it is necessary to verify the dexterity of the optimized mechanism. According to Equation (8), the global dexterity of the optimized mechanism can be calculated as 0.173 by using MATLAB R2018b. The dexterity distribution within the maximum cross-section of the workspace of the mechanism is shown in Figure 16. It can be seen that compared with the dexterity of the original mechanism (see Figure 4), the dexterity of the optimized mechanism is reduced to a certain extent. It indicates that the proposed method of optimizing the scale parameters of the 3-PSS flexible parallel micromanipulator to maximize the first-order frequency of this mechanism with the specific inscribed circle radius as the constraint is effective.

The comparison of the scale parameters of the micromanipulator, kinematic and dynamic performance before and after optimization, is given in Table 5. It is shown that the first-order natural frequency of the optimized mechanism increased by 31.07% compared with the original mechanism, which shows the effectiveness of the optimization method. The workspace of the optimized mechanism increased by 31.93% (The symbol “↑” in Table 5 indicates increase), however, the global dexterity of the optimized mechanism is reduced. It indicates that the workspace of the optimized mechanism is enlarged, but at the cost of a certain reduction in global dexterity. Therefore, in the optimization process, trade-offs must be made when faced with multiple kinematic and dynamic performance requirements. For example, if the global dexterity is more concerned than the workspace, then in the constraints (Equation (18)) one might consider choosing a smaller inscribed circle radius of the maximum cross-section of the workspace in exchange for higher global flexibility. Appendix A gives the kinematic and dynamic performances of the 3-PSS flexible parallel micromanipulator after optimization with a different inscribed circle radius as the constraints and the results confirm this rule (Table A1).

## 5. Conclusions

Based on the kinematics and dynamics of the mechanism, this paper proposes two optimal design schemes for the 3-PSS flexible parallel micromanipulator according to different application requirements and conditions. The first is called progressive optimization design, in which the scale parameters (*l* and *r_a_*) are firstly optimized to maximize the workspace, combining the constraints of the minimum global dexterity of the mechanism. Then, the minimum thickness *t_s_* and the cutting radius *R_s_* of the flexure spherical hinge are further optimized for minimizing the required driving forces, combined with constraints of the minimum first-order natural frequency of the mechanism and maximum stress of the flexure spherical hinge during the movement of the mechanism. The second is called synchronous optimization design, in which the scale parameters (*l* and *r_a_*) are optimized to maximize the first-order natural frequency of the mechanism, combined with the constraints of a certain inscribed circle radius of the maximum cross-section of the workspace, the maximum stroke of the selected piezoelectric stages, and the maximum ultimate angular displacement of the flexure spherical hinge. A comparison of the kinematic and dynamic characteristics of the original and optimized mechanism demonstrated the effectiveness of both optimization methods.

The advantage of the progressive optimization method is that both the workspace and the driving forces are optimized and the minimum requirements for global dexterity and first-order natural frequency are ensured. Thus, multiple kinematic and dynamic characteristics of the mechanism are taken into account during the optimization process. However, this optimization method needs to optimize the mechanism scale parameters followed by the structural parameters of the flexible spherical hinge in two steps, which is relatively complicated. Especially when the structure of the flexible spherical hinge is inconvenient to change, the employment of this method is limited. The advantage of the synchronous optimization method is that only the scale parameters of the mechanism need to be optimized without changing the structural parameters of the flexible spherical hinge. The optimization process takes only one step and the process is relatively simple. However, this optimization method only optimizes the first-order natural frequency of the mechanism under the premise of the requirement of a certain working space, and does not take the requirement of global dexterity and driving forces of the mechanism into consideration. Therefore, it is suggested that the optimal design scheme be reasonably selected according to different design requirements and the application of the mechanism.

## Figures and Tables

**Table 1:** Dimension parameters and material characteristics of the micromanipulator.

Parameter	Value	Parameter	Value
*r_p_* (mm)	25	*θ_l_* (°)	60
*r_a_* (mm)	45	*E* (Gpa)	200
*l* (mm)	65	*ν*	0.3
*t_s_* (mm)	1	*ρ* (g/cm^3^)	7.85
*R_s_* (mm)	2.5		

**Table 2:** Optimization results with the weight factors varying from 0.1 to 0.9.

The Weight Factors α	Structural Parameters of Flexure Spherical Hinge (mm)		Mass (kg)	The Bending Stiffness of the Hinge (N/m)	Maximum Absolute Value of Driving Force *F_bM_* (N)
	*t*_s_	*R_s_*			
0.1	0.8	1.054	0.1310	4.744	1.8397
0.2	0.8	1.584	0.1379	3.788	1.7304
0.3	0.8	2.073	0.1449	3.279	1.7303
0.4	0.8	2.292	0.1482	3.109	1.7481
0.5	0.8	2.294	0.1483	3.107	1.7483
0.6	0.8	2.294	0.1483	3.107	1.7483
0.7	0.8	2.294	0.1483	3.107	1.7483
0.8	0.8	2.294	0.1483	3.107	1.7483
0.9	0.8	2.294	0.1483	3.107	1.7483

**Table 3:** Optimization results with the weight factors varying from 0.21 to 0.29.

The Weight Factors α	Structural Parameters of Flexure Spherical Hinge (mm)		Mass (kg)	The Bending Stiffness of the Hinge (N/m)	Maximum Absolute Value of Driving Force *F_bM_* (N)
	*t*_s_	*R_s_*			
0.21	0.8	1.633	0.1385	3.726	1.7272
0.22	0.8	1.682	0.1379	3.788	1.7249
0.23	0.8	1.731	0.1399	3.611	1.7233
0.24	0.8	1.781	0.1406	3.556	1.7225
0.25	0.8	1.829	0.1406	3.557	1.7223
0.26	0.8	1.878	0.1420	3.456	1.7228
0.27	0.8	1.926	0.1427	3.410	1.7238
0.28	0.8	1.976	0.1434	3.363	1.7255
0.29	0.8	2.024	0.1441	3.321	1.7276

**Table 4:** Comparison of the structural parameters of flexible spherical hinge and dynamic performance before and after optimization.

Parameters	Before Optimization	After Optimization
Structural parameter (mm)	*t_s_* = 1, *R*_s_ = 2.5	*t_s_* = 0.8, *R*_s_ = 1.829
Total mass (kg)	0.1534	0.1406
Bending stiffness of hinge *k_bm_* (N/m)	6.5276	3.557
Ultimate angular displacement (°)	2.625	2.4739
Driving force *F_bM_* (N)	2.6329	1.7223
Natural frequency (Hz)	75.67	57.54

**Table 5:** Comparison of the scale parameters of micromanipulator, kinematic and dynamic performances before and after optimization.

Parameters	Before Optimization	After Optimization
Inscribed circle radius (μm)	307	400
Scale parameters (mm)	*l* = 65, *r_a_* = 45	*l* = 50, *r_a_* = 36.92
Volume of workspace	Original	↑ 31.93%
Global dexterity	0.2287	0.173
Natural frequency (Hz)	56.16	73.61

## Data Availability

Not applicable.

## Appendix A. The Scale Parameters of Micromanipulator, Kinematic, and Dynamic Performance of 3-PSS Flexible Parallel Micromanipulator after Optimization with Different Inscribed Circle Radius as Constraints

The scale parameters of micromanipulator, kinematic, and dynamic performance of 3-PSS flexible parallel micromanipulator after optimization with inscribed circle radius varying from 100 μm to 500 μm are as follows (The symbol “↑” and “↓” indicate increase and decrease, respectively):

**Table A1:** Comparison of the scale parameters of micromanipulator, kinematic and dynamic performances before and after optimization with different inscribed circle radius.

Parameters	After Optimization			
Inscribed circle radius (μm)	100	200	300	500
Scale parameters (mm)	*l* = 50, *r_a_* = 60.3	*l* = 50, *r_a_* = 46.82	*l* = 50, *r_a_* = 40.53	*l* = 50, *r_a_* = 62.22
Volume of workspace	↓ 67.46%	↓ 33.27%	↓ 1.03%	↑ 62.22%
Global dexterity	0.7045	0.3428	0.2309	0.1408
Natural frequency (Hz)	77.25	74.29	73.80	73.95
